# Accuracy of Non-Invasive Prenatal Testing for Duchenne Muscular Dystrophy in Families at Risk: A Systematic Review

**DOI:** 10.3390/diagnostics13020183

**Published:** 2023-01-04

**Authors:** Luca Zaninović, Marko Bašković, Davor Ježek, Ana Katušić Bojanac

**Affiliations:** 1 Scientific Centre of Excellence for Reproductive and Regenerative Medicine, School of Medicine, University of Zagreb, Šalata 3, 10000 Zagreb, Croatia; 2 School of Medicine, University of Zagreb, Šalata 3, 10000 Zagreb, Croatia; 3 Department of Obstetrics and Gynecology, University Hospital Centre Zagreb, Petrova 13, 10000 Zagreb, Croatia; 4 Children’s Hospital Zagreb, Ulica Vjekoslava Klaića 16, 10000 Zagreb, Croatia; 5 Department of Histology and Embryology, School of Medicine, University of Zagreb, Šalata 3, 10000 Zagreb, Croatia; 6 Department of Transfusion Medicine and Transplantation Biology, University Hospital Centre Zagreb, Kišpatićeva 12, 10000 Zagreb, Croatia; 7 Department of Medical Biology, School of Medicine, University of Zagreb, Šalata 3, 10000 Zagreb, Croatia

**Keywords:** non-invasive prenatal testing, cell-free DNA, Duchenne muscular dystrophy, dystrophin gene, single gene disorder, X-linked disease, relative haplotype dosage, relative mutation dosage

## Abstract

Background: Methodological advancements, such as relative haplotype and relative mutation dosage analyses, have enabled non-invasive prenatal diagnosis of autosomal recessive and X-linked diseases. Duchenne muscular dystrophy (DMD) is an X-linked recessive disease characterized by progressive proximal muscular dystrophy and a high mortality rate before the age of twenty. We aimed to systematically present obtainable data regarding a non-invasive prenatal diagnosis of DMD and provide a comprehensive resume on the topic. The emphasis was given to the comparison of different available protocols and molecular methods used for fetal inheritance deduction, as well as their correlation with prognostic accuracy. Methods: We searched the Scopus and PubMed databases on 11 November 2022 and included articles reporting a non-invasive prenatal diagnosis of DMD in families at risk using relative dosage analysis methods. Results: Of the 342 articles identified, 7 met the criteria. The reported accuracy of NIPT for DMD was 100% in all of the studies except one, which demonstrated an accuracy of 86.67%. The combined accuracy for studies applying indirect RHDO, direct RHDO, and RMD approaches were 94.74%, 100%, and 100%, respectively. Confirmatory results by invasive testing were available in all the cases. Regardless of the technological complexity and low prevalence of the disease that reduces the opportunity for systematic research, the presented work demonstrates substantial accuracy of NIPT for DMD. Conclusions: Attempts for its implementation into everyday clinical practice raise many ethical and social concerns. It is essential to provide detailed guidelines and arrange genetic counseling in order to ensure the proper indications for testing and obtain informed parental consent.

## 1. Introduction

The identification of cell-free fetal DNA (cffDNA) in maternal plasma in 1997 by Lo et al. set the scene for an immense development of techniques used for the non-invasive diagnosis of a wide range of fetal genetic disorders [1]. In addition to the widespread utilization of cell-free DNA (cfDNA) based non-invasive prenatal screening tests (NIPT) for aneuploidies, the detection of single gene disorders came into scope in recent years [2]. Duchenne muscular dystrophy (DMD) (OMIM# 310200) is an X-linked recessive inherited disease caused by a mutation in the gene encoding dystrophin (OMIM* 300377). The most characteristic features are progressive proximal muscular dystrophy and pseudohypertrophy of the calves. Progressive myofiber degeneration commonly involves the myocardium as well. It affects 1 in 3500 newborn males. The onset usually occurs in early childhood before the age of three and patients rarely outlive the age of twenty [3,4,5]. The development of NIPT techniques for Duchenne muscular dystrophy as an X-linked disease presented a substantial challenge as a consequence of high levels of maternally derived mutant alleles that outnumber potentially mutated alleles of fetal origin in maternal plasma. This issue has been resolved by the invention of molecular techniques that take into consideration the relative quantities of mutant and wild-type alleles [6,7]. These tests are primarily intended for pregnant women with DMD in their family history and therefore are considered diagnostic with no need for further confirmation by invasive testing [8]. Still, there are many concerns regarding their application, the utmost being an insufficient assessment of their validity and clinical utility. Given the low prevalence of DMD, appropriate validation of commercially available assays remains challenging. The answer may lie in large collaborative multi-institutional studies [9]. Furthermore, in the absence of formal guidelines for the implementation of the abovementioned tests into clinical practice, prompted by the opportunity for market expansion, many pharmaceutical companies began to offer these tests as part of NIPT single-gene disorder panels to the previously unselected population of pregnant women. Several ethical issues have also been identified regarding genetic counseling, pressure to undergo testing, and decisions surrounding the termination of pregnancy [7]. There are several published reviews regarding NIPT for monogenic diseases in general, but none specifically focus on Duchenne muscular dystrophy diagnosis.

With this systematic review, we intend to present obtainable data from published reports and provide a comprehensive and up-to-date resume on the topic. Our objective is a comparison of different available protocols, more precisely cohort selection processes and molecular methods used for the analysis to exhibit their correlation with an accuracy of DMD diagnosis using NIPT. 

## 2. Materials and Methods

### 2.1. Study Design and Search Strategy

The study was performed according to the Preferred Reporting Items for Systematic Reviews and Meta-Analyses (PRISMA) statement.

On 11 November 2022, we searched the PubMed and Scopus databases. The search was performed using a combination of the keywords or their equivalents, including: „diagnos*”, „screen*”, „test*”, „prenatal*, „non-invasiv*”, „cell-free DNA”, „cfDNA” and „Duchenne”, combined with Boolean operators „AND” and „OR”. There were no limits applied to the search strategy.

### 2.2. Eligibility Criteria

In order to be included, the report had to provide quantitative information on the accuracy of NIPT. We restricted our selection to English-language articles only and those regarding singleton pregnancies. Reports observing too small a sample, that is less than five pregnant women, were excluded [10,11,12,13].

### 2.3. Screening Process and Critical Appraisal

After the removal of duplicates, a study selection process was run in two stages. Firstly, two authors independently assessed records and discussed inconsistencies until consensus was obtained on which articles to screen full-text. After that, the same two researchers in a pair screened selected articles and made an agreement on which reports to include. If necessary, a third researcher was consulted to make the final decision. No automation tools were used in the selection process.

### 2.4. Data Collection Process and Risk of Bias Assessment

The data were collected from eligible studies by two independent authors. The extracted data were compared and any discrepancies were resolved by consulting a third author to make the final decision. There was no unclear information and therefore no need to get in touch with the authors of the selected reports. 

The important data items of interest were the characteristics of the study, such as country, characteristics of the study design (retrospective/prospective), the number of families at risk enrolled, characteristics of pregnant participants (age, weeks of pregnancy), family members whose blood samples were collected, type of the mutation, molecular method used for the cfDNA analysis, accuracy of mutation detection in the fetus, and invasive method used to confirm the results. Only studies confirming by invasive testing or clinical follow-up were eligible. Outcomes regarding the accuracy of the non-invasive tests conducted were pooled and reported as an overall score for every of the three main methodological approaches. We anticipated that individual reports would lack some of the data of minor importance, such as the number of informative SNPs analyzed or the coverage of the sequencing method used in the study.

The risk of bias in the included studies was assessed using the revised Cochrane Risk of bias tool. Two review authors estimated the risk for six specific domains in each of the primary studies. Any discrepancies in judgments were resolved by discussion until a consensus was reached. 

The review protocol was registered with the International Prospective Register of Systematic Reviews (PROSPERO, ID377592).

## 3. Results

### 3.1. Search Results

**Figure 1:**
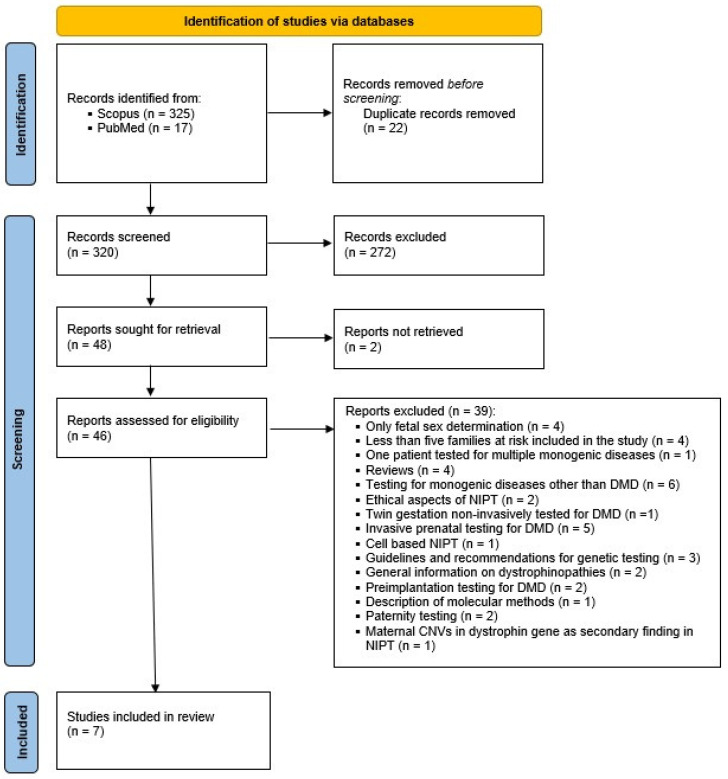
PRISMA flow diagram.

Overall, 342 records were collected by database searching. After the exclusion of duplicates, 320 remaining articles were screened based on titles and abstracts. In this step, 272 articles were excluded resulting in 48 reports intended for full-text assessment. For 2 of them, full-text reports could not be retrieved and 39 of them did not meet the criteria and were consequently excluded, leaving 7 study reports appointed for inclusion in the systematic review (Figure 1).

### 3.2. Patient Characteristics and Acquisition of Samples

Studies included in the review gathered families with a history of Duchenne muscular dystrophy (Table 1). One study conditioned enrollment on a confirmed molecular diagnosis of DMD in addition to available reference samples [14]. Another study included only families that had a previous child from their first pregnancy affected with DMD [3]. Four studies that attempted to analyze cfDNA by the indirect relative haplotype dosage (RHDO) method, obtained blood samples from the mother, proband, and father (if available) [14,15,16,17]. In cases where the proband was not available, Young et al. collected referent samples from the previous unaffected male child, other affected male relatives, or unaffected maternal grandfather [14]. The study by Chen et al. obtained only maternal plasma samples to examine prenatal detection of DMD by direct RHDO analysis [5]. A study by Jang et al. used the same technique but utilized data from their previous study while obtaining additional maternal and proband samples from one more family at risk [10,18]. Zhao et al., for their relative mutation dosage analysis, collected maternal and probands’ blood samples [3]. All the participants underwent genetic counseling and provided informed consent. Only cases of singleton pregnancies were enrolled. Consanguineous families were excluded from the research because they lack several informative single nucleotide polymorphisms required to obtain RHDO analysis [14].

### 3.3. Types of Dystrophin Gene Mutations

Dystrophin, or the DMD gene, encodes a muscle protein of the same name whose mutant variant is the cause of Duchenne muscular dystrophy. It is located on Xp21.2-p21.1, or more precisely, X:31,119,222-33,339,388 according to GRCh38 [19]. It consists of 89 exons [17]. Included studies analyzed single or multi-exon deletions, exon duplications, and point mutations [5,10,14,16,17,18]. A study by Zhao et al., in addition to point mutations, examined only small insertions and deletions, as large aberrations cannot be detected in cfDNA by relative mutation dosage technique [3,17]. All of the reports state the exact breakpoint or nucleotide change genetic coordinates for the presented cases.

### 3.4. Molecular Techniques Used for the Analysis

Two main analytic techniques are used for non-invasive prenatal diagnosis of X-linked diseases. They are designed to overcome the obstacle of identifying maternally inherited fetal alleles in the presence of an excess of maternal cfDNA in the plasma sample. They are based on relative haplotype and mutation dosage analysis [20,21,22]. RHDO leverages parental DNA haplotyping information and concludes the inheritance of the fetus by comparison of their relative ratios in cfDNA [23]. Included studies performed RHDO on targeted genomic regions in relative proximity to the DMD gene. Single nucleotide polymorphism linkage analyses were performed to determine the inheritance of the gene of interest [8,21,24].

Four studies conducted indirect haplotype phasing using proband DNA (Table 1). Capture enrichment was designed to selectively enrich target regions of chromosome X and the DMD gene, as well as sex determination loci in chromosome Y [14,15,16,17]. A study by Young et al. also designed the SNP-probe library to exons adjacent to SMN1/SMN2 and CFTR genes as they investigated several monogenic diseases in one reaction [14]. Furthermore, genomic DNA was extracted from blood samples of proband and parents and sonicated into fragments of approximately 200 bp. After being barcoded by adapter ligation and amplified by PCR, the fragmented gDNA and cfDNA libraries were used for target region capture. Post-capture libraries underwent massively parallel sequencing. Sequencing reads of gDNA were aligned to a human reference genome (hg19, GRCh37) followed by duplicate removal and variant calling to obtain SNP counts. Haplotype phasing captures the imbalance between two haplotypes in cfDNA obtained from a maternal plasma sample. Only maternal haplotyping is required for relative haplotype dosage analysis for DMD as an X-linked disease. Sequence data of families were used to construct two maternal haplotypes—one linked to the mutant allele and the other to the wild-type allele. Haplotype phasing was performed by sequencing heterozygous informative SNPs which were then grouped in statistically significant blocks and associated with one of the maternal haplotypes to calculate which one of them was overrepresented in cfDNA [14,15,16,17]. Chen et al. and Xu et al. used the Hidden Markov model to make this estimation [16,17]. In a study by Kong et al., quality control was performed. When the number of alleles linked to either mutant or wild type was less than ten, consanguineous marriage was supposed and invasive testing was suggested [15]. Because of the relatively high frequency of recombination events in the dystrophin gene of up to 12%, their probability was predicted in all four studies (Table 1) [14,15,16,17].

Although these types of tests are most frequently used in NIPT for monogenic diseases, they are not applicable in situations when proband and/or paternal DNA samples are unavailable. The solution lies in the technique of direct haplotype phasing which requires only maternal DNA material. Two studies explored this approach [5,18]. Maternal high-molecular-weight genomic DNA was partitioned into oil-enclosed gel beads. Each of the beads contained unique, distinguishable oligonucleotide barcodes that bonded to the gDNA molecule and fragmented it to produce short-tagged DNA fragments that formed a library for targeted linked-read sequencing. After sequencing, short reads were linked based on barcode tags and aligned to the region of the chromosome they originated from. Furthermore, reads with different barcodes were linked by overlapping SNP alleles. Formed haplotype blocks were finally aligned to a human reference genome (hg19, GRCh37). Maternal plasma cfDNA was prepared into libraries and enriched with capture SNP probes in the region adjacent to the DMD as a gene of interest. Heterozygous SNPs were then associated with either mutant or wild-type DMD alleles. Moreover, a relative haplotype dosage assessment was performed in order to reconstruct fetal genotypes and predict possible recombination events [5,18,25].

Zhao et al. used a relative mutation dosage approach that bases the prenatal diagnosis on the ratio calculation of mutant and wild-type alleles in maternal plasma cfDNA. This technique requires the construction of case specific-probes, therefore proband and maternal DNA samples were analyzed in order to identify the causative family-specific mutation. In one case, the mutation was caused by a deletion located in exon 12 of the DMD gene and it was necessary to map the breakpoints. Probes for PCR reaction were designed to cover the region between exons 11 and 13. Primers that did not amplify were considered to be located in the deleted region. Further analysis was conducted by a new technology called cell-free DNA barcode-enabled single-molecule test (cfBEST). Unique molecular identifiers (UMI) were ligated to cfDNA. The labeled cfDNA pre-library was split into two parts which were then amplified in three rounds and final sequencing libraries underwent additional massive parallel sequencing. Following bioinformatics refinement, the reads were aligned to a human reference genome, as in the rest of the studies, and the alleles were counted by relative mutation dosage analysis. This was the first application of cfBEST assay for the diagnosis of diseases caused by exon deletions [3].

### 3.5. Confirmatory Testing

All studies conducted confirmatory testing. Three of them obtained samples solely by chorionic villus sampling [3,5] or amniocentesis [17]. Most studies selected one of these sampling methods depending on how advanced the pregnancy was [15,16,18]. Kong et al. performed CVS on affected and amniocentesis on unaffected fetuses or carriers determined by NIPT [15]. In addition to CVS, Young et al. used postnatally obtained samples of cord blood or products of conception in cases of miscarriage and pregnancy termination [14]. Isolated genomic fetal DNA was analyzed by PCR followed by Sanger sequencing or MLPA [3,5,14,15,16,18]. Xu et al. applied the linkage-analysis method investigating microsatellites linked to the DMD gene in order to determine whether the fetus inherited a mutant or wild-type allele [17].

### 3.6. Study Outcomes

The presented studies explored the accuracy of detecting dystrophin gene mutations caused by deletions, duplications, and point variants using NIPT technology. Kong et al., in their report, demonstrated that regions of exons 8–12 and 44–55 are hotspots for dystrophin gene mutations, as that is where they located 83.2% of analyzed duplications and 76.9% of deletions [15]. All of the studies demonstrating RHDO technology identified gene variants of interest in maternal DNA samples, therefore, no de novo mutations were included in the analyses.

Studies in which targeted massively parallel sequencing technology followed by indirect haplotype phasing was applied demonstrated the lowest combined accuracy [14,15,16,17]. Young et al. reported results for not only DMD but other autosomal recessive monogenic diseases. They included 30 families at risk in their study, but NIPT by RHDO was unable to generate reportable results in four cases. In two cases this was caused by recombination events adjacent to the mutation variants of interest, while in the other two the analysis failed due to persistently low fetal fraction. Two cases showed suboptimal results—one of them also secondary to low fetal fraction, but the likely result of the fetus being affected by the disease was later confirmed by invasive testing. The other uncertain result case was derived from complex consanguinity within the family that significantly reduced the number of informative SNPs necessary for the RHDO. However, the result predicting a healthy fetus was also confirmed to be correct by invasive testing. Out of 13 cases in which the fetus was considered to be affected by a mutation, in eight of them the mother was proven to be the carrier while in the other five, germline mosaicism was suspected. Only two of these families opted for invasive testing, one of them receiving positive results. The outcomes of the other three cases were not available at the time of publication [14]. The other three studies using the abovementioned technology, however, showed a 100% accuracy, as all of the haplotypes constructed by RHDO were in concordance with the invasive testing results in absence of any false positive or negative results [15,16,17]. Kong et al. reported that in one case, an additional maternal blood draw was necessary because of a low fetal fraction below the quality control threshold of 1%. Nevertheless, the sample obtained two weeks later enabled sufficient diagnostic accuracy. In contrast to fetal fraction, all samples met the criteria regarding sequencing depth quality control. The Bayes factor used to predict inherited maternal haplotype showed substantial accuracy and correctly classified all 21 of included cases. Four of them were affected males, nine fetuses were female carriers, and the rest of them did not inherit the mutant variant of the dystrophin gene. As we emphasized in the previous example, recombination events can greatly affect prediction accuracy. In this study, they were noticed in two cases, but as their breakpoints were located about 1.30 M and 0.59 M away from the gene variants of interest, the number of informative SNPs was still adequate for RHDO analysis [15]. Another study revealed four normal non-carrier females, six healthy males, four female carriers, and three affected males out of 17 referrals. The mean achieved duplication rate, fetal fraction, 20x coverage, and capture specificity were 29.39%, 9.80%, 98.83%, and 51.60%, respectively [16]. The targeted sequencing technique by Xu et al. demonstrated a mean depth of 28.05 and coverage of 95.91% in the targeted region. By the analysis of 2590 to 7556 heterozygous informative SNPs, they correctly classified all of the referred cases—five male-affected fetuses, one female carrier, and two unaffected female fetuses. The verification of the results was carried out through invasive testing and fetal haplotype construction based on the trio strategy. The concordance ratio between maternal SNPs reached over 99.98% [17]. The combined accuracy of the indirect haplotype phasing technique was 72/76 or 94.74% (Table 1) [14,15,16,17].

Studies that exhibited linked-read sequencing—direct haplotyping method—demonstrated a 100% validity as well. The mean coverages of maternal gDNA deep-targeted sequencing were 561× and 676×, respectively. The average N50 phase-block lengths were 741.6 kb and 42.4 kb [5,18]. Although the N50 phase-block reported by Jang et al. was significantly smaller, the phasing results were sufficient for the RHDO calculation and correctly identified all four affected and one unaffected male fetus. During the analysis, a significant shift in the read fraction between samples obtained at 8 and 12 gestation weeks from the same mother was noticed. This was indicative of a recombination event and proven by comparing adjusted haplotype phasing results to the fetal genotype constructed from invasively obtained tissue [18]. Additionally, they demonstrated that, in their previous study, in which they applied an indirect haplotype phasing method, Yoo et al. incorrectly predicted a recombination event that occurred in the proband and not the fetus itself [10,18]. Chen et al. correctly identified two female carriers, two affected males, and nine normal fetuses without the mutant gene. As a consequence of an insufficient number of informative heterozygous SNPs in some cases, the DMD gene was not entirely adequately covered but this did not include the areas where variants of interest were located and therefore did not affect results [5].

Zhao et al. applied relative mutation dosage-based cfBEST and correctly diagnosed all five fetuses—one unaffected male, three carrier females, and one female with both wild-type alleles. While using this technique, prior knowledge of exact breakpoints and point mutations was mandatory. One of the mutations found in the proband was not detected in maternal gDNA, indicating that it was either a de novo mutation or the result of maternal germline mosaicism [3].

### 3.7. Risk of Bias in Primary Studies

**Figure 2:**
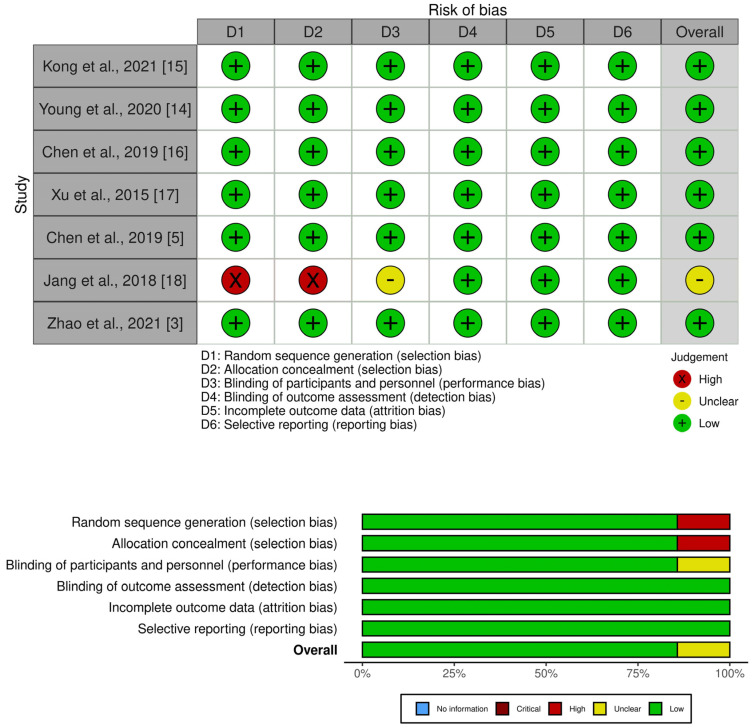
Summary of risk of bias in primary studies [3,5,14,15,16,17,18].

Due to the specific manner of studies evaluating diagnostic methods and non-invasive prenatal testing, in particular, the allocation sequence into affected/carrier/normal groups was established subsequently—after the validation of NIPT results by invasive testing methods [5,14,15,16]. Two studies blindly performed NIPT in parallel with invasive prenatal diagnosis [3,17]. Taking into consideration that DMD shows no characteristic clinical nor ultrasound or biochemical findings during pregnancy, participants and personnel were unable to predict the genetic status of the fetus. This way, the possibility for selection, performance, and detection bias introduction was minimized. Jang et al. used sequencing data from their previous study, nevertheless, they did not apprise whether the personnel conducting the recombination event detection and haplotype prediction were familiar with beforehand identified fetal genotypes. Having said that, it is unlikely that their potential knowledge of the results interfered with the outcome, as the detailed protocol was followed, as well as preset cutoff values applied, while interpreting linked-read sequencing data and predicting fetal genotype by direct haplotype phasing [18]. Our systematic review focuses on the deduction of DMD gene status. Mentioned outcome data were available for all the participants in all of the primary studies except in five cases for which the causes of incomplete results were thoroughly described [5,6] (Figure 2).

### 3.8. Limiting Factors

There are many challenges regarding the development and accuracy estimation of non-invasive testing assays for prenatal DMD diagnosis. The rarity of the condition complicates sample acquisition and handling. A guarantee of accuracy and reliability is essential for the implementation of diagnostic tests into clinical practice. The construction of a quality control protocol is necessary for the surveillance of the diagnostic process and accreditation purposes [26]. Parameters that have to be persuaded are the minimum standard of fetal fraction, sequencing read depth, and a number of informative SNPs. The earliest recommended blood collection with regard to obtaining sufficient fetal fraction samples is 7^+0^ gestation weeks [15]. The frequency of informative SNPs depends on ethnicity which may introduce bias [18]. Increasing one of the limiting parameters might compensate for the others, such as in the report by Zhao et al., in which higher fetal fraction values of 10.2% and 13.06% made up for minor sequence read numbers than those initially required [3]. Some of the studies were constructed in in silico computer simulations in order to evaluate assay performance. They calculated that a minimum of 40 SNPs is required to achieve 99% accuracy in cases with a fetal fraction less than 5%. When the sequencing depth was at least 200x, the accuracy of the test was close to 100% [16]. Even in the cases in which the fetal fraction was as low as 1%, the substantial number of 200 SNPs and 500x sequencing depth ensured assay sensitivity of over 95%. Therefore, the interaction of three factors must be taken into consideration while designing the study protocol [15,16].

Another limiting factor is the accuracy of locating recombination disruption, as it has an immense impact on predicting the accuracy of haplotype analysis in cases where the mutation variant is adjacent to breakpoints. Along with low fetal fraction, it is the most common cause of suboptimal or inconclusive results [14,17]. The recommendations should also consider filtering criteria, taking into account possible sequencing errors, the bias introduced due to library construction, and reference genome mapping efficiency, as well as fetal fraction estimation. Cases that do not meet the criteria should be reported as inconclusive and referred to invasive testing [25]. Unlike non-invasive prenatal screening for aneuploidies and copy number variants that require invasive confirmatory testing, NIPT for monogenic diseases is considered diagnostic because there are no reported placental mosaicism cases for single gene disorders [2].

Even though today NIPT for monogenic diseases is intended for singleton pregnancies only, Kong et al. reported the application of relative haplotype dosage technology for DMD testing in a dizygotic twin gestation [27].

## 4. Discussion

Until recently, the gold standard for prenatal diagnosis of Duchenne muscular dystrophy implied invasive tissue sampling by CVS or amniocentesis followed by PCR and Sanger sequencing and/or MLPA technique in order to identify potential DMD gene mutations. These procedures bear a risk of miscarriage or stillbirth with an incidence of 0.1–1.3% [28,29,30]. In recent years, since its first reported application in 2014 by Xu et al., cfDNA-based NIPT for DMD has shown immense advancements [17]. Performance of these types of tests is possible as early as 7 weeks of gestation, which is significantly earlier than for CVS [15]. Nevertheless, there are many challenges regarding the selection of referred pregnant women, including the development of molecular techniques used for the analysis, in addition to evaluation of accuracy along with the implementation of set tests into everyday practice [26]. NIPT for DMD is intended for pregnant women with a positive family history or known carriers [25]. All included studies followed these instructions throughout the process of cohort gathering. Still, the reality of clinical practice today is different and market entropy with multiple single-gene disorder panels offered to unselected, not predisposed, populations of pregnant women takes its place.

Two main methods for non-invasive DMD mutation detection in fetus include relative haplotype and relative mutation dosage. Relative haplotype dosage (RHDO) analysis deduces fetal mutation status by calculating relative ratios of maternal haplotypes associated with mutant and wild-type alleles in cfDNA isolated from maternal plasma [23]. In the targeted approach, routinely applied for single-gene disorder identification, only relative ratios of regions adjacent to the gene of interest are being determined. The RHDO procedure does not require the knowledge of family-specific mutation and the construction of mutation-specific probes in order to perform SNP linkage analysis [21,24,31,32]. Haplotype phasing of maternal DMD alleles may be performed in an indirect or direct manner. Indirect haplotype phasing, along with maternal, also requires paternal and proband or other family member’s genomic information, and therefore is not applicable in cases when they are not available, e.g., during the first pregnancy [18]. Exceptionally, Kong et al. performed an indirect analysis in cases lacking paternal samples, but this introduced a decrease in the accuracy of assay performance in differentiating female fetuses who are carriers from the ones with both wild-type alleles [15]. With direct haplotype phasing, fewer capture probes are being used and a smaller number of samples are analyzed per case, but this comes with a downside of necessity for more sophisticated equipment and consequently higher cost. Relative haplotype dosage methodology eliminates the need for specific mutation identification and enables simultaneous analysis of multiple gene loci, as well as a number of patients’ samples in a single reaction. Still, it is important to keep in mind that RHDO is not applicable for cases of de novo mutations or in occasions of maternal germline mosaicism [18,25,33]. Recombination event adjustment prior to haplotype imbalance estimation is pivotal considering the high recombination rate in the DMD region of up to 6–10% [34,35,36,37]. The direct haplotyping method illustrates a clear advantage over the indirect as it is capable of determining whether a recombination event occurred in the proband or in the fetus. The indirect method, on the other hand, requires a higher number of recombination adjustments, which leads to the accumulation of possible calculation inaccuracy. This is evident from the comparison of two reports which analyzed the same genetic data, but Yoo et al. predicted the recombination event to occur in the fetus by indirect haplotyping method; while later, the corresponding event failed to be identified by Jang et al. using direct haplotyping, which indicates that the event has actually already occurred in the proband [10,18]. Hence, when breakpoints of the recombination event are in proximity to the gene variant of interest, the confirmation of results by invasive testing is necessary [5].

Relative mutation dosage (RMD) assays, on the other hand, require the manufacturing of family-specific probes, as they deduce the fetal inheritance by calculating the ratio of mutant and wild-type alleles directly, regardless of their parental origin [25,38]. This methodology circumvents haplotype construction, which significantly reduces expenses. Unlike RHDO, it is suitable for the detection of de novo mutations and the ones inherited due to maternal germline mosaicism. Diagnostic procedures by these assays do not require recombination event assessment [3,18]. However, RMD is not the methodology of choice for the detection of mutations caused by large deletions or duplications [17].

Although NIPT enables earlier and safer diagnosis, attempts for its implementation into everyday clinical practice raise many ethical and social concerns. Primarily, the routinization of the process without meeting the criteria for patient referral. It is important for NIPT to be offered through programs in which specialized healthcare professionals provide comprehensive genetic counseling and encourage informed parental choice [2]. For families at risk, NIPT shortens the uncertainty period and reduces anxiety. It also provides parents with information to either make preparations before delivery or to consider the possibility of pregnancy termination. The development of precise recommendations and protocols by leading international organizations in prenatal care is essential for the appropriate implementation of these tests into clinical practice [26]. 

## 5. Conclusions

Non-invasive prenatal testing for Duchenne muscular dystrophy using cfDNA analysis is technologically demanding. In addition, the low prevalence of the disease reduces the opportunity for systematic research in order to assess assay validity. Even so, the presented studies demonstrate exceptional accuracy regardless of the selected methodological approach and encourage optimistic expectations for future clinical utilization.

## Figures and Tables

**Table 1:** Studies included in the systematic review.

Study	Country	Type of Study	Number of Families at Risk	Blood Samples			Fetal Fraction	Gestational Weeks	Molecular Method	Number of Informative SNPs	Coverage	Recombination Event Estimation	Confirmation Testing	Accuracy
				Maternal	Paternal	Proband’s								
Kong et al., 2021 [15]	China	Prospective cohort	21	Yes	When available	Yes	1.87–11.68%	7^+3^–18^+0^	Targeted MPS, RHDO (indirect)	1511 (DMD region) 203 (X chromosome) 213 (autosomes)	98×–563× (gDNA), 165×–490× (cfDNA)	Yes	CVS, amniocentesis (Sanger sequencing, MLPA)	100% (21/21)
Young et al., 2020 [14]	United Kingdom	Retrospective cohort	30	Yes	Yes	When available	approximately 1–18%	>8	Targeted MPS, RHDO (indirect)	-	>200×	Yes	CVS, cord blood, POC (Sanger sequencing, MLPA)	86.67% (26/30)
Chen et al., 2019 [16]	China	Prospective cohort	17	Yes	Yes	Yes	3.61–16.97%	11^+1^–26^+6^	Targeted MPS, RHDO (indirect)	3965 (flanking region of DMD)	152× (gDNA), 248× (cfDNA)	Yes	CVS, amniocentesis (targeted capture sequencing, MLPA)	100% (17/17)
Xu et al., 2015 [17]	China	Prospective cohort	8	Yes	Yes	Yes	3.52–22.67%	17–22	Targeted MPS, RHDO (indirect)	1243 (DMD region)	20× (gDNA)	Yes	Amniocentesis (microsatellites-based linkage analysis)	100% (8/8)
Chen et al., 2019 [5]	China	Prospective cohort	13	Yes	No	No	6.20–18.50%	11^+2^–25^+4^	Targeted linked-read sequencing, RHDO (direct)	2261 (DMD region) 1704 (flanking region of DMD)	329×–697× (maternal gDNA)	Yes	CVS (targeted capture sequencing)	100% (13/13)
Jang et al., 2018 [18]	South Korea	Retrospective cohort	5	Yes	No	Yes	4.10–9.25%	6^+5^–17^+1^	Targeted linked-read sequencing, RHDO (direct)	700–1000 (DMD region)	692× (maternal gDNA)	Yes	CVS, amniocentesis (Sanger sequencing)	100% (5/5)
Zhao et al., 2021 [3]	China	Prospective cohort	5	Yes	No	Yes	3.00–14.70%	11–12	RMD—based cfBEST	109	695–3476 total unique reads	no	CVS (Sanger sequencing, MLPA)	100% (5/5)

SNP—Single Nucleotide Polymorphism, DNA—Deoxyribonucleic Acid, gDNA—Genomic Deoxyribonucleic Acid, cfDNA—Cell-free Deoxyribonucleic Acid, CVS—Chorionic Villus Sampling, MLPA—Multiplex Ligation-dependent Probe Amplification, POC—Products of Conception, cfBEST—Cell-free DNA Barcode-Enabled Single-Molecule Test, MPS—Massively Parallel Sequencing, RHDO—Relative Haplotype Dosage, RMD—Relative Mutation Dosage.

## Data Availability

The data that support the findings of this study are available upon request from the corresponding author.
